# The Role of Blood–Brain Barrier Disruption in Epilepsy: Mechanisms and Consequences

**DOI:** 10.3390/neurolint18010001

**Published:** 2025-12-22

**Authors:** Elena Suleymanova, Anna Karan

**Affiliations:** Department of Molecular Neurobiology, Institute of Higher Nervous Activity and Neurophysiology, Russian Academy of Sciences, Butlerova 5A, 117485 Moscow, Russia; akartar.n@gmail.com

**Keywords:** blood–brain barrier (BBB), epilepsy, epileptogenesis, tissue-type plasminogen activator (tPA), TGF-β signaling, neuroinflammation

## Abstract

The blood–brain barrier (BBB) is essential for maintaining cerebral homeostasis, and its dysfunction is increasingly recognized as an active driver of epilepsy. This review explores the mechanisms by which BBB disruption contributes to seizures and the development of chronic epilepsy. Potentially epileptogenic insults, including traumatic brain injury, stroke, and status epilepticus, induce acute and often persistent BBB leakage. This breach permits the extravasation of serum albumin, which activates transforming growth factor-beta (TGF-β) signaling in astrocytes. This cascade leads to astrocytic dysfunction, impaired potassium buffering, neuroinflammation, and synaptic remodeling, collectively fostering neuronal hyperexcitability. Furthermore, BBB disruption facilitates the infiltration of peripheral immune cells, amplifying neuroinflammation and propagating a pathologic cycle of BBB damage and seizure activity. BBB damage is mediated by multiple processes, including the activation of the plasminogen activation (PA) system. Furthermore, these processes of BBB disruption and neuroinflammation provide a shared pathological basis for neuropsychiatric disorders like depression and anxiety, which are common comorbidities of epilepsy, through shared mechanisms of neuroinflammation and neurovascular unit (NVU) dysregulation. BBB dysfunction can also contribute to the resistance to antiepileptic drugs. Finally, we discuss the therapeutic potential of stabilizing the BBB as a viable strategy for developing disease-modifying therapies for epilepsy.

## 1. Introduction

The function of the CNS significantly depends on the microenvironment around the communicating neurons and glial cells. The barrier between the blood and this microenvironment plays an essential role in maintaining the homeostasis in the neural tissue [1,2]. The blood–brain barrier (BBB) is an active barrier between the cerebrovascular system and brain tissues. The blood–brain barrier is mainly composed of the endothelial cells connected by tight junctions, but its function is also supported by perivascular astrocytes, pericytes, microglia, and neurons. Together they form a neurovascular unit (NVU) and regulate the brain’s extracellular environment [3]. The BBB functions as a precise regulator of ionic balance through the integrated activity of specific ion channels and transporters, which is critical for effective synaptic signaling. The BBB also keeps separate the central and peripheral pools of neurotransmitters and macromolecules, protects the CNS from neurotoxic substances in the blood, and regulates the transport of nutrients and metabolites necessary for the functioning of the neural tissue [1].

Under pathologic conditions, the disruption of BBB due to degeneration of tight junctions in the endothelium, changes in the ion and macromolecule transport, pathologic angiogenesis, altered cerebrovascular perfusion, extravasation of immune cells and albumin, and inflammation contribute to the pathological cycle of neuronal and synaptic dysfunction, leading to disease progression [4]. Dysfunction of the BBB disrupts the physiological crosstalk between the periphery and the brain, exerting direct effects on virtually all neural cells. Such BBB dysfunction can both trigger and sustain seizures, thus being a cause and a consequence of epileptic activity, which makes the BBB an important link in the pathophysiology of epilepsy [5].

Epilepsy is one of the most common neurologic disorders, which is characterized by persistent, abnormal neuronal activation resulting in recurrent, typically unprovoked epileptic seizures [6]. Epilepsy is a disorder with complex etiology and in some cases can be induced by various brain insults—such as traumatic brain injury (TBI), stroke, infection, or prolonged seizures—initiating a cascade of molecular and cellular events that lead to the development of spontaneous seizures during a process, which is referred to as epileptogenesis [7]. Multiple pieces of evidence from studies on epilepsy patients and animal models demonstrate that the BBB is compromised in the epileptic brain. Moreover, BBB disruption accompanies potentially epileptogenic brain insults, including TBI, stroke, inflammatory response, and status epilepticus (SE) [8,9].

The plasminogen activation (PA) system, comprising proteases, inhibitors, and receptors, regulates the proteolytic conversion of plasminogen to active plasmin—a process fundamental to fibrinolysis—and is now established as a critical modulator of diverse cerebral functions [10]. The components of the PA system, and in particular tissue-type plasminogen activator (tPA), have been found to modulate the functions of astrocytes, neurons, microglia, and pericytes under physiologic and pathologic conditions, and participate in the regulation of BBB permeability [3,10]. It has been suggested that the PA system components participate in the pathogenesis of epilepsy by the regulation of BBB permeability [3,11].

Stress-related disorders, including depression, anxiety, and PTSD, are particularly common in epilepsy patients, with prevalence of these disorders much higher than in the general population [12,13]. BBB damage leads to a disruption of homeostasis in the brain tissues and changes in neuronal function, which can contribute to the development of neuropsychiatric disorders [14,15]. Seizures induce BBB disruption leading to hyperexcitability, neurotoxicity, and neuroinflammation, which, in turn, can be a potential link with comorbid neuropsychiatric disorders [16].

In this review, we explore how BBB disruption affects the components of NVU during potentially epileptogenic brain insults and epileptogenesis, and how increased BBB permeability translates to the development of spontaneous seizure activity. We also review the role of the PA system in BBB damage and its consequences, as well as the cascade initiated by the extravasation of serum albumin and the subsequent activation of transforming growth factor-beta (TGF-β) signaling and facilitation of peripheral immune cell infiltration in response to BBB leakage. We also explore the possible involvement of BBB dysfunction in the pathogenesis of neuropsychiatric disorders and resistance to antiepileptic drugs, which are often observed in epilepsy patients. Finally, we discuss the BBB as a promising therapeutic target for anti-epileptogenic and disease-modifying strategies.

## 2. BBB Permeability Changes in Response to Epileptogenic Brain Insults and Seizures

Clinical studies have shown that ictal activity during a seizure is followed by transient BBB opening, which can be detected on CT and MRI scans [17,18]. Potentially epileptogenic brain insults such as TBI and ischemic stroke induce acute BBB damage, and evidence also suggests an association between perivascular inflammation and BBB dysfunction in non-acquired focal epilepsies, though most findings have been made from patients with intractable epilepsy undergoing surgical resection of epileptic focus [19,20,21]. At the same time, it has been demonstrated that TBI leads to a sustained increase in BBB permeability in patients with post-traumatic epilepsy [22]. In the case of ischemic damage, it is supposed from animal studies that ischemia-induced BBB damage has biphasic dynamics: an early reversible BBB opening coincides with a decrease in perfusion and delayed irreversible associated with neuroinflammatory response [23]. Human studies confirm that early BBB damage associated with mild ischemia can be reversible if perfusion is recovered [24]. Status epilepticus (SE), including new-onset SE, when refractory seizures appear in patients without a history of epilepsy or other apparent cause, induces changes in BBB permeability detected on MRI [25,26,27]. As it clearly follows from clinical data, practically any potentially epileptogenic brain insult is accompanied by at least a transient increase in BBB permeability, which, depending on the parameters of the insult, may further remain as sustained BBB damage.

MRI studies of epilepsy patients often demonstrate signal abnormalities corresponding to BBB damage [5]. The dynamic contrast-enhanced MRI (DCE-MRI) in patients with intractable temporal lobe epilepsy (TLE) has shown multiple sites of BBB damage in the brain of patients with and without hippocampal sclerosis [28]. BBB permeability increases peri-ictally—within a few hours after the seizure onset [29]. At the same time, BBB leakage in patients with drug-resistant epilepsy is persistent and observed inter-ictally [30,31]. Notably, a recent study has demonstrated a reduction in BBB damage in TLE patients after a successful surgical resection of the epileptic focus compared to pre-surgery MRI findings, while in the patients with break-through seizures, no restoration of the BBB has been found [32].

BBB damage found on MRI in patients with generalized and secondary generalized seizures is correlated with CSF levels of metalloproteinase-9 (MMP-9) [33]. MMP-9 is a member of the family of MMPs, which play a crucial role in the regulation of extracellular matrix (ECM) remodeling [34]. MMP-9 has been found to participate in BBB damage in a number of pathologic conditions, including stroke [35], traumatic brain injury [36], and epilepsy [37,38,39]. Moreover, upon investigation, serum levels of MMPs and other ECM and BBB-associated proteins have been found to increase in patients with epilepsy, particularly in patients with drug-resistant epilepsy and hippocampal sclerosis [40,41,42]. Notably, serum levels of MMP-9 have been reported to be associated with higher seizure severity and decrease after surgical removal of epileptogenic tissue [41,43]. An increased expression of MMP-9 has been also found in surgically removed brain tissues from patients with intractable TLE [41,44] and patients with focal cortical dysplasia associated with chronic intractable epilepsy [45].

BBB changes and their role in epileptogenesis and chronic epilepsy have been massively studied on animal models, particularly on models of chronic epilepsy developing after an episode of chemically or electrically induced SE. In rodents, it has been demonstrated that permeability of the BBB for dyes, like Evans blue, or radiolabeled molecules, which normally do not penetrate through the BBB, increases after the seizures leading to the accumulation of the tracers in various parts of the brain [46,47,48]. A massive increase in BBB permeability has been detected in the acute (the first 24–48 h) period after prolonged seizures during KA- or pilocarpine-induced SE [49,50,51]. An investigation of long-term MRI changes in rat brains demonstrated that the BBB damage persisted weeks after KA-induced SE when the onset of spontaneous seizures had occurred, and in this model, it was observed within the limbic system [49].

## 3. The Mechanisms of BBB Damage

The normally functioning BBB maintains the homeostasis of the central nervous system (CNS), but various damaging events, such as inflammatory disorders, ischemic stroke, TBI, and seizures, intervene in its normal functioning and disrupt its integrity. The pathophysiological processes that drive BBB impairment and disrupt its functions, however, are diverse and context-dependent [52]. Here, we focus on potentially epileptogenic insults and how they affect the components of NVU, activating the processes that may underlie epileptogenesis and seizure recurrency.

### 3.1. Endothelial Cells and Tight Junctions

As the major structural component of cerebrovascular walls, endothelial cells are essential for regulating cerebral blood flow and maintaining vascular integrity. Inter-endothelial tight junctions, primarily consisting of the proteins claudins-1, -3, -5, -12 and occludin, are the principal component of the BBB, which maintain its structure and integrity [53,54]. It is believed that claudin-5 plays the major role in the maintaining of BBB integrity under physiological and pathological conditions [28,53]. A decrease in the expression and redistribution of claudin-5 has been associated with BBB leakage after ischemic damage [55,56] and TBI [57,58]. A significant reduction in claudin-5 expression and alterations in its distribution in the temporal lobe has been found in the surgical brain tissues from TLE patients, and similar changes have been observed in the hippocampus of mice in the chronic period after KA-induced seizures [28]. Furthermore, the targeted knockdown of claudin-5 in the murine hippocampus with the adeno-associated virus (AAV) vector expressing a short hairpin RNA against claudin-5 significantly exacerbates KA-induced seizures and BBB disruption [28]. A decrease in the expression of claudin-5 and other tight junction proteins has also been reported in the pilocarpine SE model in rats, where it coincided with upregulation of MMP-2 and MMP-9. The increase in MMP expression induced by seizures in cerebrovascular endothelial cells could disrupt the BBB through the proteolytic degradation of the tight junction proteins that seal the endothelium [37]. However, the study of the expression of tight junction proteins specifically in endothelial cells in the microvasculature of TLE patients showed an increase in claudin-5 expression and a decrease in occludin expression [59].

Vascular endothelial growth factor (VEGF) is one of the signaling molecules expressed by endothelial cells, as well as by astrocytes and neurons, in response to brain insult and inflammation [60]. VEGF mediates a number of processes, including angiogenesis, vascular permeability, and cell survival [61,62,63]. Upregulation of VEGF occurs in response to ischemic damage [64,65] and after brain injury [66,67]. While upregulation of VGEF appears to be a protective mechanism directed at repairing the cerebrovascular system, restoring blood flow, and promoting cell proliferation and survival, during the acute phase after injury, VGEF can be detrimental for brain tissues via promoting BBB leakage [68,69].

Studies on human resected tissues from patients with intractable focal epilepsies have shown a significant upregulation of VEGF family proteins [70,71]. mRNA expression and protein levels of VEGFs and their receptors are increased in the temporal cortex and hippocampus of patients with intractable TLE [72,73,74,75]. An increase in VEGF-A and its receptor VEGFR-2 levels has been found specifically in the brain microvasculature of patients with both hippocampal sclerosis TLE and secondary TLE developed as a result of a lesion [59].

Activation of VEGF expression has been reported in a number of experimental models of epilepsy in rats and mice [76,77,78,79]. It has been shown that microinjections of VEGF in the mouse cerebral cortex disrupt occludin and claudin-5 and induce BBB breakdown [80]. In vitro studies have demonstrated that seizure-like events provoke rapid upregulation of VEGF (2 h after the administration of KA) and activation of VEGF/VEGFR2 signaling, which leads to subsequent vascularization and downregulation of a key protein of tight junctions, zonula occludens-1 (ZO-1) [81]. These findings suggest that VEGF signaling is involved in the mechanisms of vascular remodeling and BBB damage induced by seizures [81].

As a part of the NVU, cerebrovascular endothelial cells participate in the regulation of ion composition and control ion transport between the vascular space and brain tissues. Endothelial cells express a number of ion transporters and ion channels, including ATP-sensitive and inwardly rectifying (Kir2.1) K+ channels, Ca2+ channels, and TRPV4 (Na+ and Ca2+) channels [82,83,84], which participate in the regulation of cerebral blood flow and neuronal function [85]. Upregulation of Kir2 channels in the hippocampal granule cells has been reported in experimental TLE [86]. Recently, it has been reported that K+ channels of the Kv7 family are also expressed in cerebrovascular endothelial cells, and their expression in the brain vessels can be significantly reduced by KA-induced SE relatively early (within 24 h) after seizure onset, possibly contributing to BBB damage [85].

Activation of brain endothelial cells in response to damaging events also results in upregulation of the cell adhesion molecules ICAM-1 and VCAM-1. These molecules facilitate the infiltration of peripheral immune cells to the brain tissues and promote neuroinflammation [54].

### 3.2. Pericytes

Pericytes are a component of the BBB, which are crucial for maintaining BBB integrity and regulating vascular function [87,88] Located within the vascular basal membrane, pericytes are mural cells that ensheathe the capillary wall and make specific contacts with endothelial cells [87,89]. Pericytes express platelet-derived growth factor receptor-β (PDGFRβ), which binds its ligand platelet-derived growth factor subunit (PDGF)-B secreted by endothelial cells [90,91]. PDGF-B/PDGFRβ signaling is involved in the regulation of angiogenesis and is an essential regulator of vascular system development [92].

A number of studies have shown that pericytes are involved in the mechanisms of the development of NVU injury after a potentially epileptogenic insult, as well as a result of seizures. Prolonged seizures during SE have been found to induce the redistribution of PDGFRβ-positive cells—pericytes—in the cerebral vasculature and brain parenchyma in the hippocampus and cortex after experimental SE, which demonstrated an SE-induced increase in pericyte vascular coverage [93]. Seizure activity has been found to promote pericytosis, which occurred simultaneously with the activation of microglial inflammatory response and the formation of pericyte-microglia clusters, resulting in BBB disruption [94]. An increase in PDGFRβ mRNA and protein levels has been reported in the lesioned hippocampus during epileptogenesis and after the development of spontaneous seizures in the KA model of TLE [95].

The neuroinflammatory response of the brain to a damaging event includes the activation of pericytes and their involvement in neuroinflammatory response [96]. Early activation of pericytes has been found to contribute to increased seizure susceptibility after cortical damage in a model of TBI by the disruption of NVU function via activation of PDGFRβ signaling in pericytes and subsequent activation of microglia [97]. Pericytes express a number of signaling molecules, primarily chemokines, including chemokine CCL2 and the adhesion molecules ICAM-1 and VCAM-1, which promote the recruitment of peripheral monocytes, T cells, eosinophils, and neutrophils, and their adhesion and extravasation [96]. It has been demonstrated that brain insults, such as inflammation and stroke, induce an increase in CCL2 production by pericytes, leading to an increase in excitatory synaptic transmission and excitation in various brain regions and promoting neuroinflammation, which can further compromise the BBB [98].

### 3.3. Astrocytes

Astrocytes are the most abundant cells in the brain, and together with other glial cells, they carry out multiple functions necessary for brain development and physiology, including regulation of synapse formation and maturity, and regulation of neuronal activity [99]. In the NVU, astrocytes send their processes to the blood vessels and form endfeet, which cover the vascular wall, while other processes can extend to various targets in the brain, including neurons and neuronal synapses, positioning them as a key regulator of NVU function. An important function of astrocytes is maintaining ion homeostasis in brain tissues by buffering extracellular K+ and regulating water balance [100]. Astrocytes participate in the maintenance of the BBB; direct evidence has been obtained in experiments with astrocyte ablation in the adult brain, showing that astrocyte loss induces a reduction in the tight junction protein ZO-1 and leads to sustained BBB damage [101].

Various types of brain insults induce astrocytic response—reactive astrogliosis—that contributes to neuroinflammation and disease progression in the context of TBI, stroke, infection, and neurodegenerative disorders [102]. Astrocytic activation is also a typical finding in epileptogenic tissues of TLE patients with hippocampal sclerosis and in animal models of epilepsy, and it is associated with an increased expression of aquaporin AQP4 and decreased expression of Kir4.1 K+ channels [103,104,105]. Kir4.1 is essential for the uptake of K+ from the extracellular space following neuronal activity, preventing its accumulation that leads to hyperexcitability. The water channel AQP4 facilitates water movement across membranes and is essential for maintaining osmotic balance and supporting K+ clearance. Kir4.1 and AQP4 are critical for preventing excitotoxicity by ensuring a stable neuronal microenvironment [106]. The loss of Kir4.1 in the epileptogenic tissue of TLE patients with hippocampal sclerosis has been observed in perivascular endfeet of astrocytes [107]. AQP4, despite reports of significant upregulation in TLE, undergoes redistribution leading to its reduction in perivascular endfeet [108]. Dysregulation of astrocytic Kir4.1 and AQP4 expression leads to the disruption of water and ion balance, which can contribute to changes in neuronal function and hyperexcitability. Such changes also can affect NVU function and modulate BBB permeability, and the modulation of AQP4 expression has been demonstrated to affect BBB integrity in experimental models of brain damage [109,110]. At the same time, BBB disruption itself can induce rapid changes in AQP4 expression [111].

### 3.4. Microglia

Microglia are the principal immune cells of the brain participating in a number of processes maintaining homeostasis in the brain tissues, and they play an important role as a part of the NVU [112]. Under pathological conditions, when brain injury occurs as a result of trauma, ischemic damage, or seizures, microglia activation takes place [113,114,115]. Activated microglia can express pro-inflammatory and anti-inflammatory cytokines, including tumor necrosis factor α (TNFα), TGF-β, interleukins IL-1β and IL-10, chemokines, reactive oxygen species, nitrogen monoxide (NO), and many others depending on the phenotype [116]. Activation of microglia is well documented in human epilepsy and in experimental models of seizures [115,117,118,119]. Seizures induce rapid activation of microglia and the release of the pro-inflammatory cytokines TNF-α and IL-1β, which induce inflammatory response and activation of astrocytes and endothelial cells, resulting in the expression of a number of proteins, modification of endothelial tight junctions, and production of NO and MMPs, which lead to BBB disruption [112,120,121]. BBB dysfunction and ECM degradation, in turn, can lead to the production of factors activating microglia and microglia-mediated inflammation, demonstrating bidirectional BBB–microglia interactions in the integrated context of the NVU, where active crosstalk between microglia, endothelial cells, and other components of the NVU contribute to the progression of inflammation [122].

**Figure 1:**
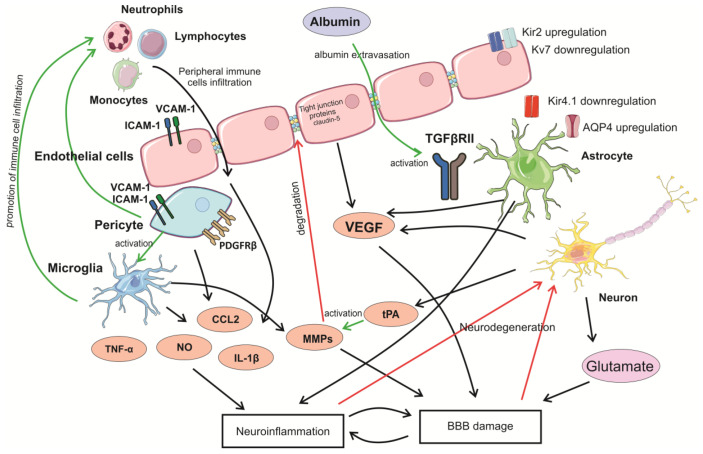
Simplified schematic representation of processes activated in the BBB after a potentially epileptogenic brain insult. Brain damage or neuronal hypersynchronization during seizures induce rapid glutamate release and alter the expression of a variety of proteins by NVU components. An increase in the expression of VEGF, MMPs, tPA, pro-inflammatory cytokines, and cell adhesion molecules promotes the disruption of BBB integrity and neuroinflammatory response. Disruption of BBB integrity and secretion of pro-inflammatory cytokines, chemokines, and cell adhesion molecules leads to infiltration of peripheral immune cells to brain tissues, which further promotes neuroinflammation. Extravasation of albumin leads to activation of TGF-β signaling. Changes in AQP4 expression and distribution and altered expression of K+ channels disrupt ion homeostasis and water balance affecting neuronal function. AQP4—aquaporin 4; CCL2—C-C motif chemokine ligand 2; ICAM-1—inter-cellular adhesion molecule 1; IL-1β—interleukin 1β; Kir2, Kir4.1—potassium inward rectifier channels; Kv7—voltage-gated potassium channel; MMP—matrix metalloproteinase; NO—nitric oxide; PDGFRβ—platelet-derived growth factor receptor β; TGFβRII—transforming growth factor β receptor II; TNF-α—tumor necrosis factor α; tPA—tissue-type plasminogen activator; VCAM-1—vascular cell adhesion molecule 1; VEGF—vascular endothelial growth factor.

The components of the NVU and processes occurring in response to brain insults are schematically represented in Figure 1.

## 4. Plasminogen Activation System and BBB Dysfunction

The PA system converts proenzyme plasminogen into its active form, plasmin, by tissue-type (tPA) or urokinase-type (uPA) plasminogen activators. The activity of this proteolytic cascade is tightly controlled by serine protease inhibitors (serpins), primarily plasminogen activator inhibitor-1 (PAI-1) and neuroserpin, which are the main physiological inhibitors of tPA [10]. Multiple studies have demonstrated that tPA can affect NVU function, cerebral flow, and BBB permeability both via its proteolytic and plasmin-independent signaling function [123].

The plasminogen activators tPA and uPA are involved in the regulation of MMP activity [124]. The MMP family includes zinc endopeptidases that target extracellular matrix (ECM) proteins and can proteolytically alter the ECM integrity and degrade vascular matrix integrity, thus contributing to BBB damage [125]. Administration of exogenous tPA has been reported to increase MMP-9 activity during ischemic damage [126], and it also mediates upregulation of MMP expression [125,127,128]. MMPs are secreted as inactive proenzymes that require activation by an extracellular protease cascade: tPA and uPA convert plasminogen into plasmin, which, in turn, proteolytically activates MMPs [129]. Besides proteolytic activation, tPA also regulates MMP activity via activating LRP1-mediated cell signaling [125,130].

Besides upregulation of MMP expression, tPA and uPA/uPAR activate a number of other LRP1-mediated signaling pathways, like NFkB-dependent expression of pro-inflammatory cytokines and the complement system, contributing to neuroinflammatory response [131], which could also contribute to BBB disruption [132].

The function of tPA as a signaling molecule is also carried out via its interaction with NMDA receptors (NMDARs). Depolarization induces binding of NMDARs by tPA, which potentiates NMDAR signaling [133]. Activation of neuronal NMDARs by tPA promotes excitotoxic damage and neuroplasticity [134,135]. tPA has been proposed as a regulator of neurovascular coupling: tPA induces cerebral blood flow in response to neuronal activation via NMDAR-mediated activation of NO synthesis [136,137]. Activation of endothelial NMDARs is known to cause cellular redistribution of occludin and an increase in BBB permeability, and it has been demonstrated that NR1 in brain endothelial cells plays a critical role in regulating BBB permeability by mediating tPA-induced signal transduction and controlling the transmigration of monocytes [138,139].

BBB permeability can also be regulated via PDGF-CC signaling. PDGF-CC is a member of the PDGF family, which can bind PDFGRα and β receptors [140]. PDGFRα signaling plays a vital role in tissue development and homeostasis, and contributes to various processes during brain development [11,141]. PDGF-CC is a homodimeric protein that is expressed in its inactive form, containing a N-terminal CUB domain, which requires activation via proteolysis [142]. The proteases tPA and uPA can activate PDGF-CC, so they can bind its receptor and promote PDGF-CC/PDGFRα signal transduction [143,144,145].

Studies on experimental models of ischemic stroke have shown that the activation of PDGF-CC/PDGFRα signaling in perivascular cells in response to ischemic damage rapidly increases BBB permeability [146,147]. The serpins PAI-1 and neuroserpin regulate the activity of tPA, so they can negatively control BBB permeability via limiting tPA activity [11]. It also has been demonstrated that activation of PDGFRα by tPA/PDGF-CC requires co-receptors, such as low-density lipoprotein receptor-related protein 1 (LRP-1). Binding of LRP-1 by tPA leads to a significant increase in BBB permeability [146,147,148].

Besides the relatively well-studied role of tPA/PDGF-CC in brain ischemic damage, the effects of tPA on BBB permeability have been implicated in the pathogenesis of TBI and inflammatory neurological disorders, such as multiple sclerosis [146,149].

The ability of tPA to increase BBB permeability has significant implications in pathophysiology and treatment of ischemic stroke. Administration of recombinant tPA to patients with acute ischemic stroke is an approved method of thrombolysis and restoration of cerebral perfusion in such patients [150,151,152]. However, treatment with tPA can cause complications, such as intracerebral hemorrhage—hemorrhagic transformation [152]. Moreover, a few studies have suggested that tPA treatment can increase the risk of development of post-stroke epilepsy [153,154]. Such risks of tPA thrombolysis in stroke have been heavily discussed, and recent studies have suggested that adequate tPA therapy applied at an appropriate therapeutic time window generally does not increase the risks of post-stroke epilepsy, and the potential epileptogenic effects of tPA are insignificant compared to the benefits of reperfusion therapy [155,156,157]. However, factors such as large size of ischemia-induced lesion, intracerebral hemorrhage, and cerebral infarction with hemorrhagic transformation clearly increase the risk of early post-stroke seizures and post-stroke epilepsy [156,158,159], so tPA may be involved in the risks of epilepsy development in the case of severe brain damage and complications of stroke. It has been demonstrated that tPA administration increased the number of neutrophils and T cells in the blood of patients with ischemic stroke and in animals with experimental stroke, which transmigrate to the brain and exacerbate BBB damage, increasing the risk of hemorrhagic transformation [150]. Notably, inhibiting endogenous tPA after recanalization is reported to attenuate brain damage and improve recovery after experimental ischemic stroke, while delayed tPA treatment, on the contrary, exacerbated the damage [160].

A significant body of existing data on the role of tPA in the regulation of BBB permeability has been obtained from stroke patients and experimental ischemia models, where exogenous recombinant tPA was applied. Less is known about the effects of the role of endogenous tPA and other components of the PA system in the regulation of BBB permeability in epilepsy. tPA and its inhibitor neuroserpin are expressed in NVU cells, most likely by neurons innervating the vascular wall and regulating vascular functions [161]. In studies on knockout mice, tPA-deficient tPA−/− mice have demonstrated decreased susceptibility to seizures [161,162], while neuroserpin-deficient Nsp−/− mice, on the contrary, have developed very severe seizures in response to KA injection [161]. At the same time, Nsp−/− mice have been characterized by a rapid loss of BBB integrity, while tPA−/− mice have shown a decrease in BBB leakage in response to KA-induced seizures [161]. Inhibition of tPA-induced PDGFRα signaling has attenuated BBB leakage and delayed the propagation of seizure activity, and astrocyte-specific PDGFRα deficiency in mice has also led to a delay in generalization of KA-induced seizures, demonstrating that tPA regulates BBB permeability and seizure spreading via the PDGFRα signaling pathway [161].

**Figure 2:**
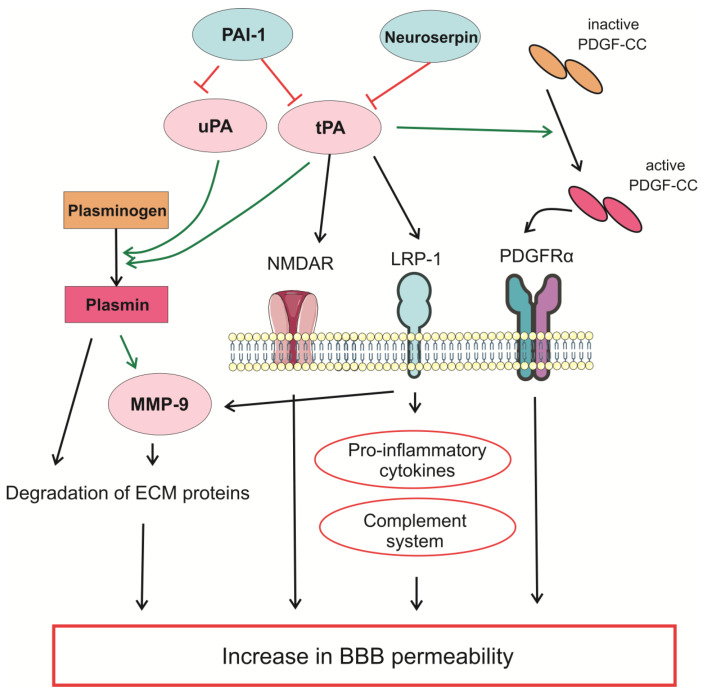
Schematic representation of how the PA system participates in the regulation of BBB permeability. tPA and uPA convert plasminogen into its active form, plasmin, which proteolytically degrades ECM proteins and activates MMPs. tPA can regulate BBB function via plasmin-independent mechanisms, such as binding NMDA receptors, activation of LRP-1-mediated increase in the expression of MMPs, pro-inflammatory cytokines, and the complement system. tPA also activates inactive form of PDGF-CC, which binds PDGFRα receptors and activates a number of processes leading to an increase in BBB permeability. Green arrows indicate activation; red arroes indicate inhibition. BBB—blood–brain barrier; LRP-1—low-density lipoprotein-related protein 1; MMP-9—matrix metalloproteinase 9; NMDAR—N-methyl-D-aspartate receptor; PAI-1—plasminogen activator inhibitor 1; PDGF-CC—platelet-derived growth factor CC; PDGFRα—platelet-derived growth factor receptor α; tPA—tissue-type plasminogen activator; uPA—urokinase-type plasminogen activator.

Figure 2 schematically shows the participation of the PA system in the regulation of BBB permeability.

## 5. BBB Damage in the Pathogenesis of Epilepsy

Seizures can affect NVU function and the integrity of the BBB via a number of mechanisms, including alteration of the basal membrane of the BBB and endothelial cells forming it, as well as changes in the neuronal, astrocyte, and pericyte function [9]. Pathologic hypersynchronization of neuronal activity during epileptic seizures leads to rapid release of glutamate, inducing changes in NVU component function that result in increased vascular permeability and BBB opening [21,163]. Rapid excessive glutamate release also occurs after the onset of ischemia and in response to trauma, such as TBI [164,165].

Altered BBB permeability, in turn, results in the extravasation of albumin and peripheral blood immune cells [9,166].

### 5.1. BBB Permeability and Peripheral Immune Cell Migration in the Pathogenesis of Epilepsy

Epileptogenic insults can damage the BBB, which is suggested to establish a pro-inflammatory microenvironment and facilitate crosstalk between the central and peripheral immune systems [167]. This increase in BBB permeability triggers the invasion of peripheral immune cells—such as neutrophils, T and B lymphocytes, and monocytes—into the brain, initiating a cascade of events that sustains neuroinflammation [9,168].

A few studies have shown the activation of peripheral T cells and changes in the CD4(+) and CD8(+) T cell profile in TLE patients, demonstrating persistent peripheral inflammation in such patients [169,170,171]. Furthermore, increased peripheral levels of pro-inflammatory cytokines are associated with drug-resistant epilepsy [169]. An increased level of IL-1β-positive CD14+ monocytes has been found in patients with drug-resistant epilepsy, and it correlated with seizure frequency [172]. The correlation between peripheral and central inflammation suggests that peripheral inflammatory biomarkers could potentially be used to evaluate neuroinflammation in drug-resistant epilepsy [173]. These clinical findings also support the existence of crosstalk between central and peripheral immune systems in epilepsy patients.

A significant challenge arises from the postulated ability of monocytes to differentiate into “microglia-like” cells, making it difficult to discriminate them from the native microglial population [174].

Peripheral blood leukocyte invasion has been detected in surgically removed tissues of TLE patients [175,176] and patients with cortical dysplasia [177,178].

The invasion of peripheral immune cells has been shown in experimental models of acquired epilepsy, including intrahippocampal KA injection, a pilocarpine SE model, and febrile seizures [176,179,180,181,182]. Potentially epileptogenic events, such as TBI and ischemic stroke, also lead to leukocyte invasion [183,184]. Neutrophil invasion in the models of ischemia and TBI has been found to contribute to an increase in MMP expression, which in turn, could contribute to the development of inflammatory response and further BBB breakdown [185,186].

The facilitation of neuroinflammatory response, neuronal injury and death, and further BBB damage by infiltrating leukocytes has been found in a KA-induced TLE model in rats [182]. Inhibition of monocyte infiltration to the brain attenuated neurodegeneration, reduced pro-inflammatory cytokine IL-1β levels, and decreased BBB damage in the KA and pilocarpine models [187]. In the rat model of electrically induced SE, the number of infiltrated CD68+ monocytes, together with perivascular CD163+ macrophages and activated microglia, positively correlated with the duration of initial SE and with the frequency of spontaneous seizures in the chronic period after SE, while T cells and dendritic cells scarcely infiltrated the brain parenchyma in this model [124,188]. In the pilocarpine-induced status epilepticus (SE) model, early phases of epileptogenesis are characterized by the infiltration of Ly6G+Ly6C+ neutrophils and the upregulation of proteins, associated with BBB dysfunction, such as MMP-3 and MMP-9 [189].

### 5.2. Extravasation of Albumin and TGF-β Signaling

One of the primary mechanisms of the input of BBB damage into the development of hyperexcitability has been established as a signaling cascade triggered by extravasation of blood serum albumin. Post-mortem immunohistochemical investigation of the hippocampus of epilepsy patients showed the presence of extravasated serum albumin and activated astrocytes [190]; albumin immunoreactivity has been found also in autopsy material and the surgically removed epileptogenic brain tissues from patients with intractable TLE [190,191].

It has been demonstrated that exposure to albumin resulting from BBB damage and to exogenous albumin application induces hyperexcitability and hypersynchronization in vivo and in vitro [192]. In experimental epilepsy models, albumin promoted seizure susceptibility both short- and long-term. It has been demonstrated that administration of albumin facilitates KA-induced epileptiform activity and recurrent ictal events in vitro [193]. Intracerebral administration of albumin increased susceptibility to KA-induced seizures during the acute period after albumin injection and reduced the after-discharge threshold in the hippocampus in the long-term period after albumin injection, demonstrating sustained increase in the neuronal excitability induced by albumin [194].

Albumin induces hyperexcitability and promotes epileptogenesis by activating astrocytes via TGF-β receptors. It has been shown that albumin accumulates in astrocytes and binds TGF-β type II receptors (TGFβRII) and activates the TGF-β signaling pathway, which regulates the expression of multiple genes leading to the induction of astrocytic activation and neuroinflammatory response [192,195]. Blocking TGF-β receptors prevents the development of albumin-induced epileptiform activity [195].

The canonical TGF-β signaling pathway is activated when the ligand binds to the transmembrane TGF-β type II receptor (TGFβRII). This activates TGFβRII, enabling it to phosphorylate the TGF-β type I receptor, also called activin-like kinase 5 (ALK5 or TGFβRI) [196,197]. Phosphorylated ALK5, in turn, phosphorylates intracellular SMAD2/3 proteins, which translocate to the nucleus and alter the transcription of a wide spectrum of genes [198,199].

TGF-β signaling is pleiotropic and context-dependent [199]. Notably, it has been demonstrated that the canonical TGF-β signaling pathway is activated in the dentate gyrus and is involved in the regulation of adult neurogenesis [198]. It also regulates migration and growth of neural progenitor cells, and is also involved in the regulation of neuronal survival via PI3K/Akt and MAPK/ERK1/2 pathways [200,201]. It has been shown that albumin activates the ALK5–SMAD2/3 pathway predominantly in astrocytes, but not in neurons, where it activates the ALK1–SMAD1/5/8 pathway [202].

The functional changes induced by TGFβR activation in astrocytes are carried out via several downstream mechanisms. Activation of TGF-β signaling leads to downregulation of Kir4.1 and accumulation of extracellular K+ and glutamate and a subsequent increase in neuronal excitability [199].

An exposure to albumin and activation of TGF-β signaling in astrocytes induces transcriptional changes leading to altered expression of ECM-related proteins and degradation of perineuronal nets around inhibitory parvalbumin interneurons [203], which could contribute to the alteration of inhibitory synaptic connections and lead to hyperexcitability. Albumin also has been shown to induce changes in neuronal potentiation, a reduction in long-term depression and increase in long-term potentiation in response to stimulation [204]. It also can promote excitatory synaptogenesis, which can be blocked by a specific ALK5/TGF-β inhibitor [205].

Another mechanism is the induction of pro-inflammatory cytokine expression. TGF-β1, via activation of SMAD2/3 pathway, induces early upregulation of pro-inflammatory cytokine interleukin IL-6 in astrocytes, which also can cause neuronal excitability and trigger epileptogenesis [206].

The involvement of TGF-β signaling in pathogenesis of epilepsy is supported by clinical data. Increased concentration of TGF-β1 has been found in the CSF of patients with drug-resistant epilepsy [207], and TGFβRIs are upregulated in the temporal lobe of such patients [208]. There is a significant increase in the expression of the components of the TGF-β canonical pathway—TGF-β1, TGFβRII, SMAD3, and Smad anchor for receptor activation (SARA)—in the surgically removed epileptogenic tissues of the temporal lobe of patients with intractable TLE [209,210].

TGF-β1 can induce the expression of MMP-9 in brain astrocytes via activation of kinase signaling cascades and activation of the NF-κB transcription factor, which contributes into the processes of ECM remodeling [211], which implicates TGF-β signaling in the regulation of ECM metabolism and neuronal plasticity.

TGF-β signaling is also a well-known factor inducing upregulation of PAI-1 [212], and it has been demonstrated that TGF-β can induce an increase in PAI-1 expression in astrocytes [213]. Upregulation of PAI-1 and increased inhibition of tPA has been suggested as a potential mechanism of the neuroprotective effect of TGF-β after a brain insult inducing rapid tPA upregulation [214]. At the same time, studies have reported that PAI-1 antagonists can have a neuroprotective effect on experimental TBI and stroke [215,216], demonstrating that TGF-β-induced increases in PAI-1 expression can also have a detrimental effect depending on the context.

## 6. BBB Dysfunction and Stress-Related Neuropsychiatric Disorders

A growing body of evidence shows that stress-induced dysfunction of the neurovascular unit and increased blood–brain barrier (BBB) permeability is a potential pathological link to psychiatric disorders like major depressive disorder (MDD), but the exact mechanisms underlying this process are not fully understood [217]. The association of BBB dysfunction with neuropsychiatric disorders has been reported in MDD patients and experimental models [218,219], as well as in experimental anxiety and PTSD [220,221,222].

Animal studies have shown that the processes and signaling pathways participating in the regulation of BBB permeability and associated with BBB damage can be also implicated in the pathogenesis of anxiety and depression. In animal models of chronic stress, NVU alterations and changes in BBB permeability have been reported to induce anxiety- and depressive-like behavior, accompanied by downregulation of the tight junction protein claudin-5 in the brain tissues [223,224].

It recently has been demonstrated that increased VEGF-A plasma and CSF levels correlated with BBB permeability in MDD patients, and inhibition of VEGF-A/VEGFR-2 signaling with a monoclonal antibody suppressed BBB dysfunction and the development of depressive-like behavior in the chronic restraint stress model of depression, which suggested an important role of VEGF-A/VEGFR-2 in the development of depression [217].

A significant body of data demonstrates that inflammation is involved in the pathogenesis of a number of neuropsychiatric disorders, including major depressive disorder (MDD), posttraumatic stress disorder (PTSD), and anxiety [225,226,227]. An increase in markers of inflammation—plasma and CSF levels of pro-inflammatory cytokines and their receptors, chemokines, and adhesion molecules—has long been reported in MDD patients [228,229,230,231], and findings from animal experiments have suggested the involvement of neuroinflammation in the development of depressive-like behavior [232,233,234]. Neuroinflammation is also reported to be implicated in anxiety and PTSD [235,236,237]. BBB is an important link in the interactions between the brain and the immune system which potentially implicates BBB damage in the pathogenesis of various disorders, associated with neuroinflammation [238]. BBB damage is associated with leukocyte infiltration to the brain, which results in amplifying and maintaining neuroinflammation [239]. Studies on animal models of depressive and anxiety disorders have shown that recruitment of peripheral monocytes to the brain can be triggered by stress and are associated with depressive-like and anxiety-like behavioral changes [240,241]. It has been demonstrated that chronic stress can induce an upregulation of VCAM-1 and ICAM-1 adhesion molecules in brain endothelial cells and promote monocyte infiltration and an increase in the production of IL-1β, which leads to the development of stress-induced anxiety [240].

Notably, the peripheral levels of TGF-β have been reported to be decreased in patients with MDD and anxiety [242,243], and in experimental models of stress-induced depression, the expression of TGF-β is downregulated in the brain of animals with depressive- and anxiety-like behaviors [244,245]. At the same time, as discussed in previous sections, the TGF-β/TGFβRII signaling pathway is involved in epileptogenesis, and its components are upregulated in TLE patients [205,209], which is very distinct from depressive and anxiety disorders. However, it has been reported that in cancer-related depression, TGF-β serum levels are increased in cancer patients with and without depression [246]. These findings suggest that TGF-β signaling is implicated differently in the pathogenesis of MDD and comorbid depression and might not be a marker of depression in the case of comorbidity, unlike MDD.

## 7. BBB Dysfunction and Resistance to Antiepileptic Drugs

Despite a large variety of antiepileptic drugs, antiepileptic treatment fails to control seizures in about 30% of patients with epilepsy, and this ratio persists despite the development of novel antiseizure medications [247]. Despite the increased permeability of the damaged BBB, its dysfunction leads to a decrease in drug penetration to the brain in epilepsy patients [9]. Since BBB is an active barrier participating in the regulation of the transport of a wide spectrum of molecules between peripheral circulation and brain tissues, BBB dysfunction may be one of the mechanisms of the development of resistance to antiepileptic drugs.

While most drugs targeting brain tissues, including antiepileptic drugs, are lipophilic and are supposed to easily cross the BBB via lipid membranes, lipid-soluble drugs often exhibit lower-than-predicted brain permeability due to active efflux by transporters at the blood–brain barrier, which efficiently limit their CNS uptake [9,248]. The transporter hypothesis suggests that the resistance to antiepileptic drugs develops as a result of overexpression of drug efflux transporter proteins, such as P-glycoprotein (Pgp) and other multidrug transporters, in the BBB in epileptogenic brain tissues [249]. Overexpressed multidrug transporters lower extracellular drug levels in the region of the epileptogenic pathology, diminishing the efficacy of antiepileptic treatment [250]. The transporter hypothesis is supported by evidence from both animal and human studies. An increase in the expression of the MDR1 gene encoding Pgp and Pgp protein expression has been found in the resected brain tissues from patients with drug-resistant epilepsy [251,252,253]. Moreover, an increased Pgp immunoreactivity has been associated with high risks of seizure recurrence in epilepsy patients after surgery for drug-resistant TLE [254]. In vivo investigation of Pgp activity using positron emission tomography (PET) with a radiolabeled Pgp substrate has shown that patients with drug-resistant TLE have higher Pgp activity in the temporal lobe compared to seizure-free patients, and higher levels of Pgp activity corresponded to higher seizure frequency [255]. PET imaging has also revealed an increase in Pgp activity in patients with different epileptogenic developmental lesions (focal cortical dysplasia and hamartoma), showing that Pgp activity increase may take place in drug-resistant epilepsy regardless of etiology [256]. Experimental studies have demonstrated overexpression of Pgp in cerebrovascular endothelial cells of rats with post-SE recurrent spontaneous seizures, who did not respond to phenobarbital, in comparison to epileptic rats responding to the medication [257]. Pgp overexpression at the BBB in the limbic structures of chronically epileptic rats has been demonstrated to reduce the local levels of phenytoin compared to that in the regions with unchanged Pgp expression [258]. In the rat kindling model, overexpression of Pgp was also reported, which led to reduced accumulation of carbamazepine and phenytoin in brain tissues [259].

Despite abundant evidence of overexpression of drug transporters associated with epilepsy, the role of these changes in the development of resistance to antiepileptic drugs is still under debate, mainly due to controversial data on the transport of epileptic drugs by drug efflux transporters [250,260]. While there is evidence that some antiepileptic drugs, such as phenytoin, phenobarbital, oxcarbazepine, lamotrigine, topiramate, and lacosamide, are likely the substrates for Pgp, others, such as valproate, gabapentin, and vigabatrin, are probably not [261].

In addition to changes in drug efflux transporter expression, it has been shown that the resistance to antiepileptic drugs is associated with increased expression of cytochrome P450 (CYP) enzymes in BBB endothelial cels, which are responsible for local drug metabolism at the BBB [262,263]. It has been demonstrated that the silencing or inhibition of nuclear glucocorticoid receptors (GRs) regulating the expression of CYP leads to a decrease in CYP function and an increase in BBB permeability for phenytoin and a reduction of oxcarbazepine metabolism, which indicates that GR and CYP may play a role in the mechanisms regulating the bioavailability of antiepileptic drugs, thus contributing to the development of drug resistance [264,265].

## 8. BBB Targeting as a Potential Therapeutic Strategy

The involvement of BBB damage in pathogenesis of multiple neurologic disorders makes it an attractive target for therapeutic interventions. A few strategies have been offered to prevent BBB damage or mitigate its harmful downstream effects, depending on the mechanism of BBB damage and signaling pathways involved.

In the context of epilepsy and epileptogenesis, the TGF-β/TGFβRII signaling pathway as a target for antiepileptogenic treatment has drawn significant attention. Experiments on animal models have shown that blocking albumin-induced TGF-β signaling by losartan prevents the development of albumin-induced seizures [202]. Losartan is angiotensin AT1 receptor antagonist, which is primarily used in the therapy of hypertension, but it has also been found to inhibit TGF-β signaling and decrease albumin-induced p-Smad2/3 upregulation and astrocytic activation [202]. Losartan also has shown anti-seizure effects in a model of audiogenic seizures and PTZ-induced kindling [266,267]. Losartan has been reported to attenuate astrocyte activation and BBB damage and inhibit spontaneous seizures in a lithium–pilocarpine SE model [268]. Interestingly, a cohort study on patients with arterial hypertension has shown that patients receiving angiotensin receptor blockers, particularly losartan, demonstrated a lower incidence of new-onset epilepsy compared to patients receiving other groups of antihypertensive medications [269].

A few studies have shown that epileptogenesis can be modified via targeting microRNAs (miRNAs). miRNAs are a group of small non-coding RNAs which perform regulatory functions during development and under pathological conditions by suppressing protein expression at the post-translational level [270,271]. It has been demonstrated that the levels of miRNAs can be altered in experimental epilepsy models and in human epileptogenic tissues [272,273,274]. The analysis of miRNAs’ expression profiles during epileptogenesis demonstrates that these alterations are associated with a number of signaling pathways, including TGF-β signaling and pathways related to ECM regulation [274,275]. Suppressing some miRNAs, including miRNA-134, miRNA-124, miRNA-135a, and some others, can inhibit seizure activity and reduce spontaneous seizure activity in experimental models of chronic epilepsy [276,277,278]. Overexpression of miRNA-132 in cultured astrocytes has been found to suppress TGF-β2 expression and reduce the expression of pro-inflammatory factors [279]. Interestingly, increased BBB permeability can be exploited for therapeutic benefit, as demonstrated in experiments with timed administration of an intravenous antisense oligonucleotide (antagomir) against miRNA-134 during a period of BBB disruption, which allowed the drug to reach the brain and exert an anti-epileptic effect [280].

Beyond their symptomatic control of seizures, some conventional antiseizure drugs have been evaluated for their potential to prevent the development of epilepsy following a brain insult [281]. Levetiracetam, an approved antiepileptic drug for treatment of various types of seizures, has been found to produce anti-epileptogenic effects in various models of seizures, including kindling, audiogenic seizures, and SE-induced chronic epilepsy [282,283,284]. Treatment with levetiracetam has been demonstrated to inhibit transient SE-induced BBB damage and prevent pro-inflammatory responses and expression of pro-angiogenic factors, and also suppress the infiltration of neutrophils in the hippocampus [189,285]. Another approved antiepileptic drug, valproate, has been demonstrated to reduce BBB damage in experimental intracerebral hemorrhage via a decrease in the expression of MMP-9 and the pro-inflammatory cytokines TNF-α and IL-6, with a simultaneous increase in the expression of claudin-5 and occludin in the brain [286]. While antiepileptogenic properties of valproate are not confirmed [287,288], its neuroprotective effects have been reported in experimental models of ischemic brain damage and TBI [289,290].

Pathways which can be potentially targeted for stabilization of BBB permeability include PDGFRα/β signaling. The tyrosine kinase inhibitor imatinib suppresses the phosphorylation of PDGFRα and PDGFRβ and inhibits these receptors [11]. Inhibition of PDGF-CC/PDGFRα signaling has been found to reduce BBB dysfunction and reduce neuronal damage associated with ischemic stroke and TBI [291,292]. Pre-treatment with imatinib has been found to delay the onset and generalization of KA-induced seizures [161]. It has also been demonstrated that pre-treatment with imatinib together with valproate reduces the severity of PTZ-induced kindling and attenuates spontaneous recurrent seizures in a pilocarpine SE model [293].

Despite the promising results from experimental studies of BBB modulation, most approaches of BBB targeting are still far from translation into clinical settings [9]. Repurposing existing drugs, such as losartan, offers many advantages, including reduced costs and timelines due to established safety profiles of existing compounds. At the same time, there are still many challenges due to complexity of the disease and requirement of multidisciplinary cooperation, which make it difficult to create an appropriate study design [294]. The dosage of medication and timing of therapeutic intervention are also very important, particularly in the case of treatment of acute, potentially epileptogenic brain insults, such as ischemic stroke, and further studies are required to obtain this data [295]. Moreover, regulation of BBB permeability is very complex and involves a large number of participants with bidirectional and context-dependent interactions. BBB damage also occurs locally and involves region-specific alterations in the processes underlying BBB dysfunction. For example, TGF-β signaling is characterized by profound complexity and diversity involving a large network of components and significant pathway crosstalk with precise spatiotemporal control [296]. This intricate regulation makes therapeutic targeting particularly challenging.

To summarize, the results of experimental studies on animal models of seizures and BBB damage show that targeting specific molecular pathways underlying BBB disruption—notably TGF-β signaling—represents a viable strategy for developing anti-epileptogenic therapies. These approaches aim to prevent the progression to chronic epilepsy by disrupting the cycle of BBB damage and neuroinflammation, contributing to hyperexcitability and epileptogenesis. However, so far, none of the studied candidates have exhibited an ability to fully prevent epileptogenesis. Moreover, there is evidence that increased BBB permeability in some cases might be beneficial for drug delivery in the therapy of potentially epileptogenic brain insults. At the same time, some processes accompanying BBB disruption may be a compensatory response to brain damage; for example, infiltration of peripheral leukocytes can have a protective effect via recruitment of B-cells to the brain, which can have protective or detrimental effects depending on the cell phenotype, timing, and microenvironment [297].

## 9. Conclusions

Evidence from both clinical studies and experimental models suggests that BBB dysfunction is involved in pathogenesis of epilepsy and can be a consequence of seizures, but also acts as a driver of epileptogenesis. Brain insults, such as traumatic injury and ischemic damage, disrupt the functions of cells constituting the NVU, leading to changes in vascular permeability and BBB leakage. BBB disruption, in turn, activates pathologic cascades of processes, including the extravasation of serum albumin and activation of TGF-β signaling, initiating astrocytic dysfunction, neuroinflammation, and synaptic remodeling, which lower the seizure threshold and promote the development of chronic epilepsy. Epileptic activity itself can initiate BBB disruption, and chronic recurrent seizures contribute to the maintaining of BBB leakage in the epileptic brain. Importantly, the compromised BBB is also involved in the pathogenesis of stress-related neuropsychiatric disorders. Neuroinflammation and dysregulation of the neuronal microenvironment caused by BBB disruption provide a shared pathological substrate for the common neuropsychiatric comorbidities of epilepsy, such as depression and anxiety. Consequently, restoring the BBB and mitigating the consequences of BBB leakage with drugs like losartan or through approaches like microRNA regulation represents a viable strategy to disrupt the pathologic cycle induced by BBB disruption, potentially providing both seizure control and alleviating comorbid psychiatric symptoms. However, there is evidence that the response of NVU components and activation of signaling pathways, as well as their timing, may differ depending on the context and type of brain insult, and are complicated by intricate, reciprocal interactions within the NVU. Therefore, future research is needed to elucidate the precise spatiotemporal details of BBB damage and NVU dysfunction across different epileptogenic contexts.

## Data Availability

No new data were created or analyzed in this study.

## Abbreviations

The following abbreviations are used in this manuscript:

AAV	Adeno-Associated Virus
ALK5	Activin Receptor-Like Kinase 5
AQP4	Aquaporin-4
ATP	Adenosine Triphosphate
BBB	Blood–Brain Barrier
CNS	Central Nervous System
CSF	Cerebrospinal Fluid
CT	Computed Tomography
DCE-MRI	Dynamic Contrast-Enhanced Magnetic Resonance Imaging
ECM	Extracellular Matrix
KA	Kainic Acid
LRP-1	Low-Density Lipoprotein Receptor-Related Protein 1
MAPK/ERK1/2	Mitogen-Activated Protein Kinase/Extracellular Signal-Regulated Kinase 1/2
MDD	Major Depressive Disorder
miRs/miRNAs	MicroRNAs
MMP	Matrix Metalloproteinase
MRI	Magnetic Resonance Imaging
NF-κB	Nuclear Factor Kappa-Light-Chain-Enhancer of Activated B Cells
NMDAR	N-Methyl-D-Aspartate Receptor
NO	Nitric Oxide
NVU	Neurovascular Unit
PA system	Plasminogen Activation System
PAI-1	Plasminogen Activator Inhibitor-1
PDGF	Platelet-Derived Growth Factor
PDGFRα/β	Platelet-Derived Growth Factor Receptor Alpha/Beta
PI3K/Akt	Phosphoinositide 3-Kinase/Protein Kinase B
PTSD	Post-Traumatic Stress Disorder
SE	Status Epilepticus
TBI	Traumatic Brain Injury
TGF-β	Transforming Growth Factor-Beta
TGFβRI/II	Transforming Growth Factor-Beta Receptor Type I/II
TLE	Temporal Lobe Epilepsy
TNFα	Tumor Necrosis Factor Alpha
tPA	Tissue-Type Plasminogen Activator
TRPV4	Transient Receptor Potential Vanilloid 4
uPA	Urokinase-Type Plasminogen Activator
uPAR	Urokinase-Type Plasminogen Activator Receptor
VEGF	Vascular Endothelial Growth Factor
VEGFR-2	Vascular Endothelial Growth Factor Receptor 2
ZO-1	Zonula Occludens-1
